# Institutional Questions

**Published:** 1900-03-24

**Authors:** 


### INSTITUTIONAL QUESTIONS.

**Isolation Hospital Staffs.**

The matron of an isolation hospital (twenty-six beds), all scarlet fever, in two blocks, administrative block, wash-house, laundry, disinfecting chamber, and garden grounds (one acre), &c., would be glad if you would give her some little idea as to what staff she should have to carry out the work, say, for fifteen patients as at present. The present staff consists of matron, caretaker (or porter), one nurse, one probationer, one general servant, and two charwomen (now resident in washhouse).—Bona fide.

*** Everything depends upon the extent to which an isolation hospital is used. The persons mentioned might be sufficient for a permanent staff if the hospital were but little used. But if males and females, and perhaps children, suffering from two or perhaps three different diseases such as scarlet fever, diphtheria, and enteric fever are to be received, it is clear that a much larger staff will be required. We do not think that a small isolation hospital should take probationers. They want fully-trained nurses. Probationers should be trained in large institutions under competent supervision. It is obvious that in an institution with only one nurse and one probationer, the probationer from the first day of her entry must at times be left in sole charge, which is wrong.

